# Measurement of sodium in soft tissue and bone in a sodium diet intervention study using *in vivo* neutron activation analysis

**DOI:** 10.1088/1361-6579/ae0dee

**Published:** 2025-10-15

**Authors:** Song Yue, Sana Tabbasum, Jolie Susan, Amy Atun, Nicole N Karongo, Valerie Mercer, Natalie Sweiss, Connie M Weaver, Cheryl AM Anderson, Linda H Nie

**Affiliations:** 1 School of Health Sciences, Purdue University, West Lafayette, IN, United States of America; 2 Herbert Wertheim School of Public Health and Human Longevity Science, University of California, San Diego, CA, United States of America; 3 School of Public Health, San Diego State University, San Diego, CA, United States of America; 4 Division of Nephrology and Hypertension, University of California, San Diego, CA, United States of America; 5 School of Exercise and Nutritional Sciences, San Diego State University, San Diego, CA, United States of America

**Keywords:** *in vivo* neutron activation analysis, sodium intake, sodium in soft tissue and in bone

## Abstract

**Objective.:**

Sodium (Na) overconsumption has been associated with hypertension risk and progression. Human soft tissue and bone are recognized as quickly and slowly exchangeable compartments for sodium storage. How such a distribution regulates blood pressure remains unknown. This study performed *in vivo* Na measurements on human subjects who underwent dietary intervention, utilizing a compact neutron generator-based neutron activation analysis system. It aimed to evaluate the performance of this innovative system for body Na assessment.

**Approach.:**

Participants were provided with low and high sodium diets. Baseline measurements were taken before each intervention feeding period, and follow-up measurements were conducted afterwards. The human hands were irradiated for 20 min, followed by 2 cycles of Na gamma ray collection. A biokinetic model was used to calculate sodium concentrations in soft tissue and bone, reflecting sodium accumulation in the two compartments.

**Main results.:**

For soft tissue, Na levels after low Na diet decreased from baseline in half of the subjects, with reductions ranging from 8% to 55%. The other half of participants exhibited relatively stable Na content. Among participants consuming high Na diet, all participants had elevated Na in soft tissue compared to those on low Na diet. By contrast, Na in bone showed no significant changes from baseline and follow-up for either dietary intervention. Bone Na concentrations ranged from approximately 1000–2000 ppm.

**Significance.:**

For the first time, Na in soft tissue and bone was measured in humans using neutron activation analysis in response to dietary interventions. This study demonstrates that *in vivo* neutron activation analysis can be used to measure Na concentration in both soft tissue and bone. It successfully detects Na alteration in soft tissue and explores the biokinetics of Na retention following dietary interventions. Measuring soft tissue and bone sodium content is a potentially useful approach to study diet and disease links affected by sodium.

## 1. Introduction

Sodium (Na) homeostasis is critical to maintaining electrolyte balance and normal organ functioning (Bernal *et al* 2023). As the primary extracellular cation Na regulates water by osmosis, hence it affects blood pressure (Noda and Matsuda 2022). Excessive salt consumption from food has been reported to be associated with higher risk of heart failure, kidney disease, and the prevalence of hypertension (Aaron and Sanders 2013). Managing Na intake can be crucial for hypertension prevention and treatment. Although pharmacological treatment is widely available, blood pressure control remains unsatisfactory in patients who receive medication (Mahmood *et al* 2019). Alongside direct medical treatments, the dietary approaches to stop hypertension (DASH) have been shown by research groups worldwide to be an effective method for hypertension prevention (Mahmood *et al* 2019, Soltani *et al* 2020). It can significantly lower medical costs while reducing complications. Although dietary guidelines were established to restrain hypertension progression, these suggestions are challenged in the clinic (Aaron and Sanders 2013, National Academies of Sciences 2019, Shim *et al* 2020, Unger *et al* 2020). Twenty-four-hour urinary sodium excretion has been the gold standard for estimating Na intake (Ma *et al* 2022). Spot urines have also been used but have produced either conflicting or inaccurate Na intake assessment (Dougher *et al* 2016). More importantly, Na accumulation not only happens in extracellular fluids but also in soft tissue and bone. The two compartments are recognized as quickly and slowly exchangeable pools for Na metabolism and are considered as more important Na storage organs (Cohen-Boulakia *et al* 1981, Mohseni *et al* 2016). However, measuring Na in soft tissue and bone presents a significant challenge, which results in limited data on Na in these storage organs. Consequently, there is a knowledge gap on how Na in these organs is associated with blood pressure. This knowledge could provide mechanistic insights for the role of Na in chronic disease and validate public policy recommendations.

Previously, ^23^Na-MRI was developed and used to map Na distribution in soft tissue (Kopp *et al* 2013, Madelin and Regatte 2013, Canaud *et al* 2019). For example, significant Na was found in skin and muscle among patients with kidney disease (Sahinoz *et al* 2021). A study also showed elevated Na could serve as biomarkers for distinguishing lipedema from obesity (Crescenzi *et al* 2018). However, the image quality was much lower compared to conventional MRI and needs to be greatly improved due to the magnetic property of Na nucleus (Zaric *et al* 2021). Additionally, Na levels in bone are difficult to assess with MRI because of the dense structure and the low concentration of Na. Given these limitations, our lab developed a compact neutron generator-based *in vivo* neutron activation analysis (IVNAA) system to quantify Na in soft tissue and in bone (Coyne *et al* 2019). The system has been designed and developed for human elemental composition determination in the past a few years (Liu *et al* 2014, 2017), offering a promising unique technique that allows non-invasive quantification of various elements in the body. It produces thermal neutrons to stimulate the production of characteristic gamma rays from research subjects, which are then detected and analyzed to identify and quantify the elements of interest. With customized shielding and experimental design, it was successfully used for manganese and aluminum measurements in humans (Liu *et al* 2018, Hasan *et al* 2020). Because of the excellent penetration of thermal neutrons, IVNAA can interact with Na directly regardless of its storage organ.

The overall research objective was to examine how dietary sodium affects body Na. In this paper, we report the first *in vivo* Na measurement in human soft tissue and bone under dietary intervention, and explore the effects of high and low dietary sodium intake on the storage and excretion of Na.

## 2. Materials and methods

### 2.1. Study population

This study recruited human subjects who volunteered to contribute to the research project at University of California, San Diego (UCSD). Individuals meeting preliminary eligibility criteria, based on self-reported responses regarding age, high blood pressure diagnosis and medication, smoking, diabetes, use of supplements, pregnancy, food-related allergies, and availability to participate, were invited to attend a screening visit. Candidates were excluded if they were determined to have pre-existing significant lipid disorder, abnormal liver or kidney function, illicit drug use, or use of medications that affect lipids or alter mineral absorption or metabolism. Written informed consent was obtained from participants. This study was approved by the UCSD institutional review board (IRB) and participating site, Purdue University, relied on this single IRB for ethical review. The approved experimental protocols comply with the principles of the Helsinki Declaration. The radiation safety was reviewed by the UCSD Human Exposure Review Committee and audited by the Environmental Health & Services Radiation Safety office at UCSD.

### 2.2. Neutron instrumentation and radiation safety

**Figure 1.**
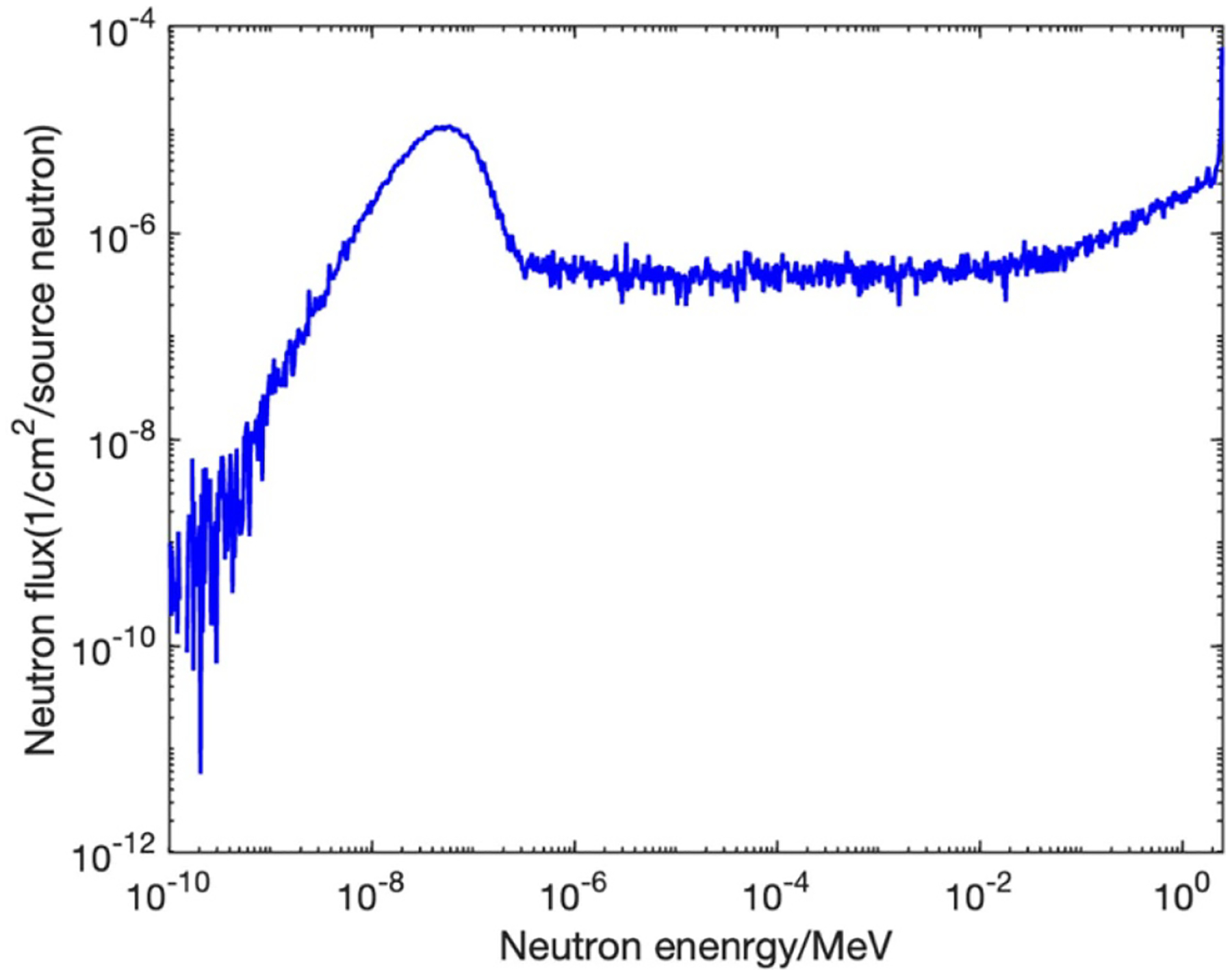
Simulated neutron energy spectrum in the irradiation cave.

The IVNAA system is based on a deuterium-deuterium neutron generator, which approximately produces 7×10^8^ neutrons s^−1^ with an average energy of 2.45 MeV (Liu *et al* 2014). These high energy neutrons will not interact with Na and only contribute to radiation dose. Hence, they were slowed down by moderator (Liu *et al* 2017). Given by Monte Carlo simulation, the moderated neutron spectrum in irradiation cave is shown in figure 1. The thermal neutrons produced after moderation will activate ^23^Na by thermal neutron capture reaction and emit characteristic Na gamma rays. The gamma rays are then collected by a model GMX 100P4–95-A GAMMA-X High Purity Germanium (HPGe) detector.

An electronic dosimeter was used for radiation monitoring and quality assurance. The measured radiation dose to the irradiated hand averaged 2.5 rem and 0.25 rem for neutrons and photons, respectively, during experiments. The estimated total body effective dose for a reference man is 0.02 mSv, which is far below 1 mSv annual limit for the general public.

### 2.3. System calibration

Similar to our previous studies (Liu *et al* 2018), human bone and soft tissue equivalent phantoms were fabricated based on International Commission on Radiological Protection recommendations (ICRP) (Liu *et al* 2014). Na was doped to phantoms to create calibration lines. The bone phantoms were prepared using a mixture of calcium sulfate, manganese (II) nitrate, ammonium chloride and sodium nitrate (NaNO_3_), and magnesium sulfate and sealed by vacuumed plastic bags. Soft tissue phantoms consisted of water doped with varying amount of NaNO_3_. The mass of soft tissue phantom is around 62 g. To ensure uniform distribution and prevent contamination, all chemical compounds were first dissolved in distilled water before being incorporated into the phantom matrix. The irradiation protocol of phantoms was identical to that used in human measurements. Calibration lines were generated based on count rates.

### 2.4. Human irradiation and signal acquisition

**Figure 2.**
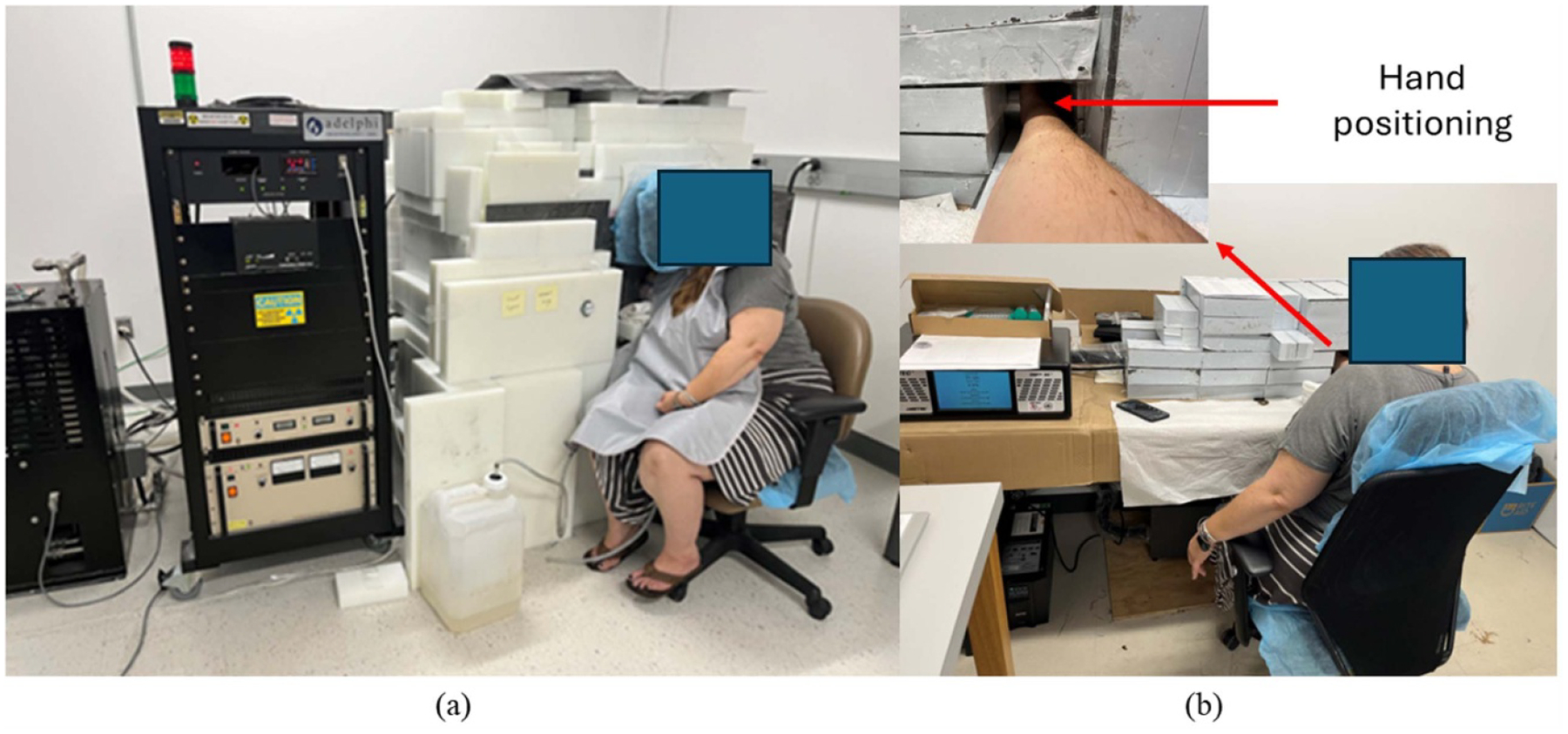
A scene of human experiments (a) *In vivo* irradiation setup. (b) Gamma ray detection.

The human hand was inserted into constructed irradiation cave, shown in figure 2(a). Na is activated following equation (1). The two gamma rays, with energies 1368 keV and 2754 keV, were collected by the HPGe detector (figure 2(b)). Participants were informed that they should hold their hand still to reduce movements during the gamma ray detection. The human hand positioning in this procedure is the same as phantoms in system calibration.

**Figure 3.**
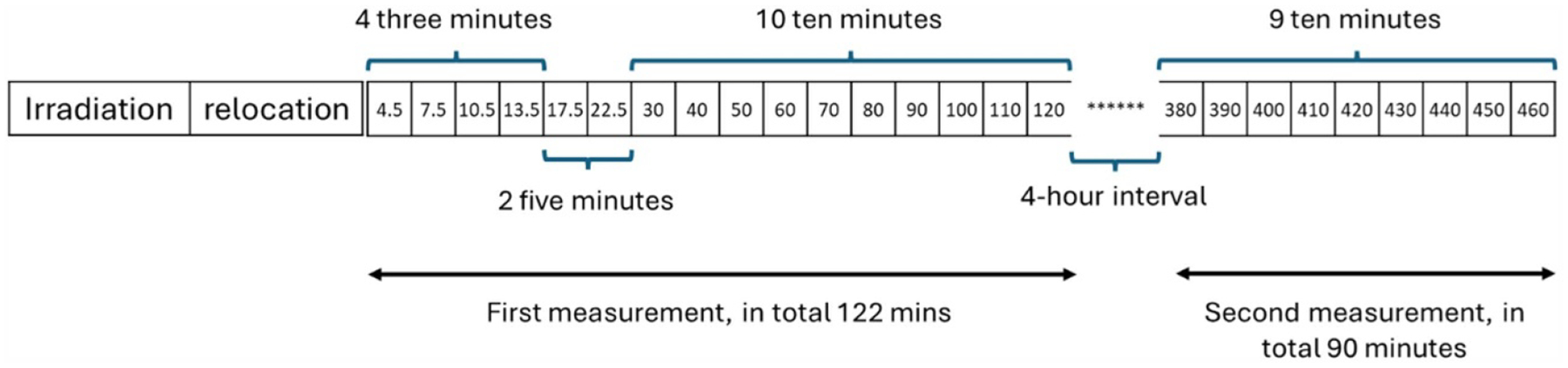
Experimental time scheme for irradiation and measurement.

To distinguish Na from soft tissue and bone compartments, the gamma rays were collected at multiple time periods. As mentioned above, soft tissue and bone are two pools for Na, which means they have significantly different metabolic rates. Sequential gamma ray collection can capture this behavior, thus offering a way to distinguish where Na comes from. In this study, the irradiation time is 20 min. Following that, there is a three-minute period to move the participant from the irradiation site to the HPGe detector. The first measurement began immediately afterward and included 16 time intervals, in total 122 min. A four-hour rest and meal period was followed to allow Na to release from the soft tissue. A second measurement was conducted right after to monitor signals primarily from bone, consisting of 9 time intervals over a total duration of 90 min. Figure 3 illustrates the entire time scheme. The intensive sampling using shorter time intervals at the beginning of first measurement is to catch the change of the Na signals in quickly exchangeable compartment, e.g. soft tissue.


(1) thermal neutron+N23a→N24a+γ(1368keVand2754keV).

### 2.5. Human dietary intervention

**Figure 4.**
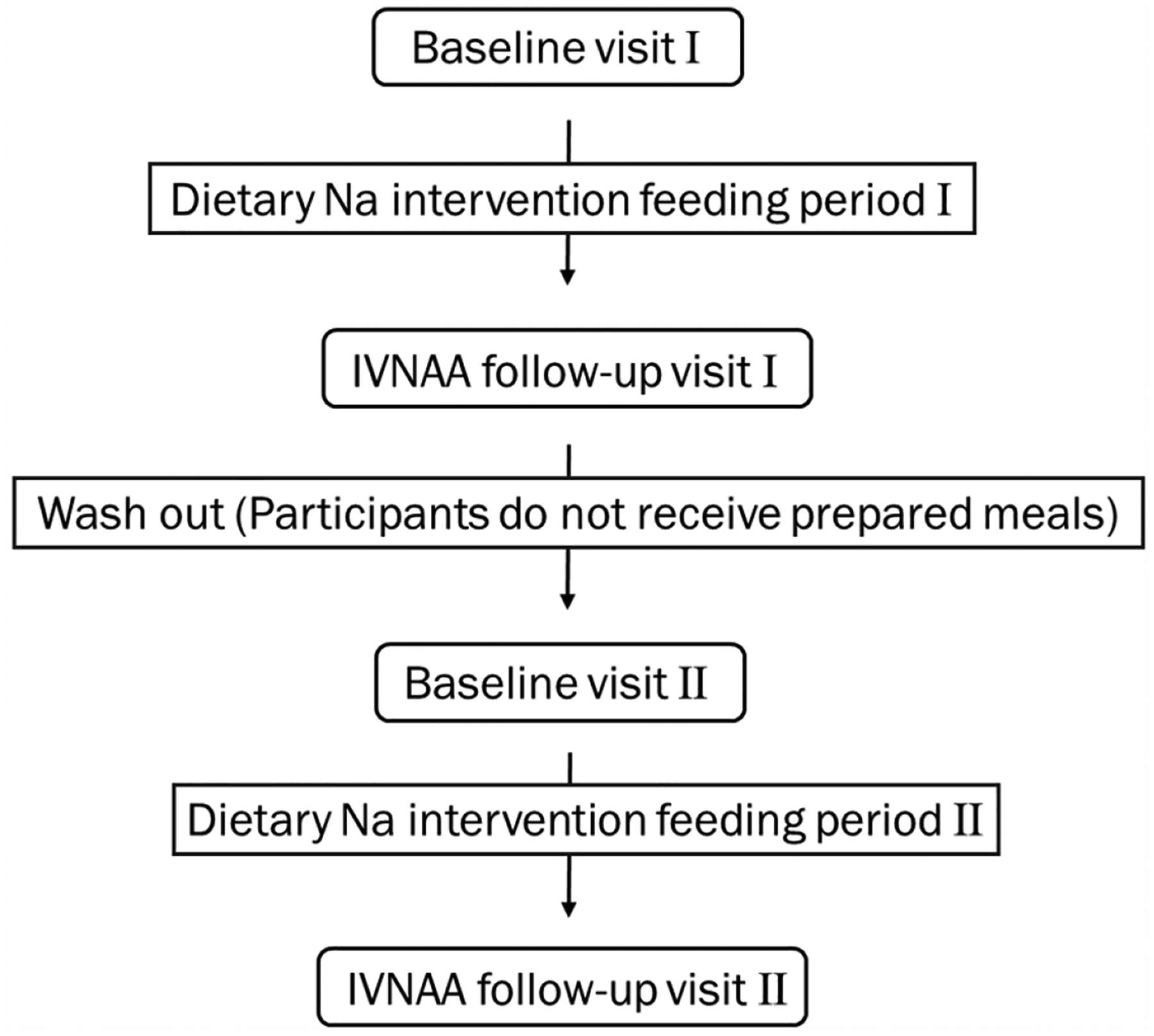
Dietary intervention design.

The two dietary interventions were: (1) a ‘high’ sodium diet; and (2) a ‘low’ sodium diet. For individuals consuming 2200 calories d^−1^, Na levels in meals and drinks were 3400 mg d^−1^ (148 mmol d^−1^) for the high Na diet, representing the highest Na intake used for adults in the DASH study (Sacks *et al* 2001) and the estimated average daily intake in the United States; and 2300 mg d^−1^ (100 mmol d^−1^) for the low Na diet, representing the upper limit of current recommended daily intake (Dietary Guidelines for Americans 2015). The cross-over dietary intervention design is summarized in figure 4. First, each participant was invited to participate in a baseline Na measurement. Then, the order in which participants received the two dietary interventions (2 weeks) was randomized. At the end of the first dietary intervention, participants completed a follow-up visit. Following that, an approximately one-week washout separated the two dietary intervention periods of the study. Baseline and follow-up measurements were repeated for the second feeding plan. Some participants completed both intervention periods, while others only completed one due to budget constraints. In total, seven human subjects participated in the protocol.

### 2.6. Data analysis

As described above, IVNAA does not provide spatial information for the elements. To differentiate Na in bone and soft tissue, we utilized the distinct biological exchange of Na in these two compartments. ICRP recommended internal dosimetry for occupational intake radionuclides calculations (Paquet *et al* 2017). The exponential function is a good approximation to mimic biological clearance of radioisotopes. By modifying this theory, a biokinetic model was developed to decompose signals and determine Na in different compartment (equation (2)). ${\dot{C}}_{1}$, ${\dot{C}}_{2}$ represent the maximum independent count rates for soft tissue and bone after irradiation. *T*_*i*_ is the total effective half-life of ^24^Na in soft tissue and in bone respectively, which can be calculated by equation (3),

(2) $$\dot{C}(t)={\dot{C}}_{1}{\text{e}}^{-\frac{\text{ln}\;2}{T_{1}}\times t}+{\dot{C}}_{2}{\text{e}}^{-\frac{\text{ln}\;2}{T_{2}}\times t}$$

(3) $$\frac{1}{T_{\text{i}}}=\frac{1}{T_{\text{biological decay}}}+\frac{1}{T_{\text{physical decay}}}.$$

It is reported that the biological decay for Na in bone is up to days or even weeks (Cohen-Boulakia *et al* 1981, Mohseni *et al* 2016). So, the biological decay can be ignored when considering Na release from the bone. The model can be further simplified by fixing *T*_2_ at the physical half of activated ^24^Na, which is 14.956 h. All other parameters can be obtained by nonlinear fitting. We chose a 4 h interval, rather than a longer one, due to scheduling constraints. Four hours is relatively short for the removal of Na from soft tissue, according to previous reports (Mohseni *et al* 2016). Consequently, the second measurement may include residual gamma counts from the soft tissue. Direct model fitting converges locally, hence gamma signal from the bone cannot be recognized properly. By setting *T*_2_ to 14.956 h manually, the two compartments can be identified without compromising accuracy. The subcomponent in equation (2) was used to determine count rate for each compartment based on integral equation (4). The corresponding calibration lines were applied to calculate concentrations,

(4) $$\dot{C}=\frac{\int^{T}{\dot{C}}_{i}{\text{e}}^{-\frac{\text{ln}\;2}{T_{i}}\times t}}{T}$$

(5) $$\frac{\dot{c}}{m\;\mu g}=\frac{\dot{C}}{M}.$$

Exact human hand-shaped calibration phantoms are not available. And there are anatomical differences among participants. This discrepancy is addressed for bone and soft tissue differently. For bone, the calibration line is normalized to simultaneously activated Calcium (^49^Ca, 3084 keV characteristic gamma ray, 8.72 min half-life), correcting for factors including geometry and hand size. For soft tissue, this correction is not applicable. Instead, count rate per Na mass was calculated from the slope of the calibration. If soft tissue phantom mass is *m* grams, the Na mass *M* in human hand can be calculated by equation (5), where $\dot{c}$ is count rate from calibration line slope, and $\dot{C}$ is the calculated soft tissue count rate from equation (4). With the same participants, the Na mass can reflect concentration change in soft tissue.

Also, we have not taken the fast exchange of Na in soft tissue during experiments into consideration, which means that the above result will be an underestimation. To account for the Na removal from the soft tissue through circulation, decay corrections were made using specific effective half life *T*_1_ for each measurement.

The original model development can be found in our previous study (Yue *et al* 2025). More details on correction and calculation were discussed.

## 3. Results

### 3.1. Calibration lines

**Figure 5.**
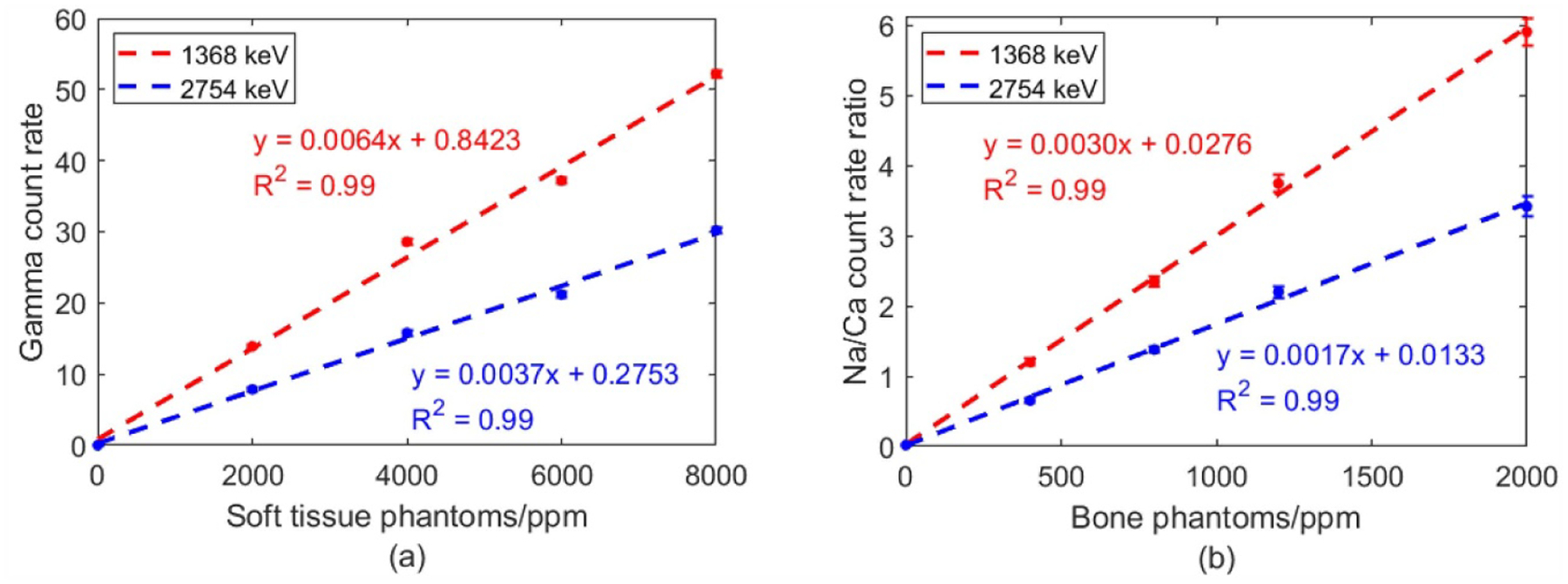
Calibration lines for *in vivo* Na quantification (a) soft tissue (b) bone.

The Na concentrations in bone and soft tissue phantoms were 0, 400, 800, 1200, 2000 and 0, 2000, 4000, 6000, 8000 ppm (1 ppm = 1 *μ*g Na/g bone), respectively. Both 1368 and 2754 keV gamma rays were analyzed, as shown in figure 5. Soft tissue calibration lines were based on count rates. While Na/Ca count rate ratios were applied to create calibration lines for bone.

### 3.2. Gamma ray spectrum of the *in vivo* measurement and Na biokinetics over time

**Figure 6.**
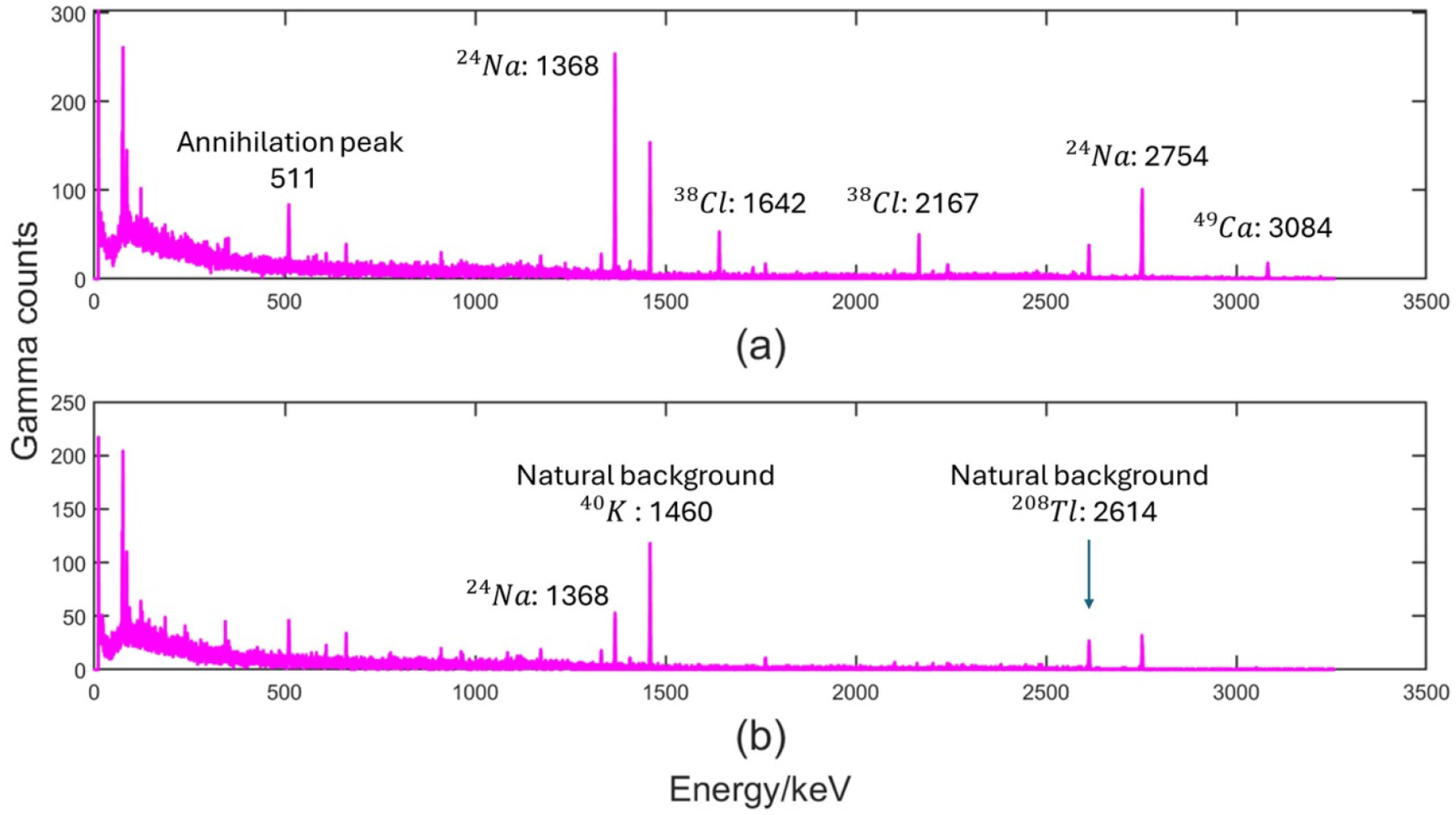
*In vivo* accumulative gamma spectrum for a subject, peaks relevant to calculations are marked (a) first 122 min measurement (b) second 90 min measurement after 4 h break.

To show the ability of IVNAA to quantitate Na in humans, cumulative spectra during two counting periods were plotted in figure 6. The total number of Na gamma ray counts decreased significantly due to physiological washout. ^49^Ca signal at 3084 keV disappeared in the second measurement because of its short physical half-life of 8.72 min.

**Figure 7.**
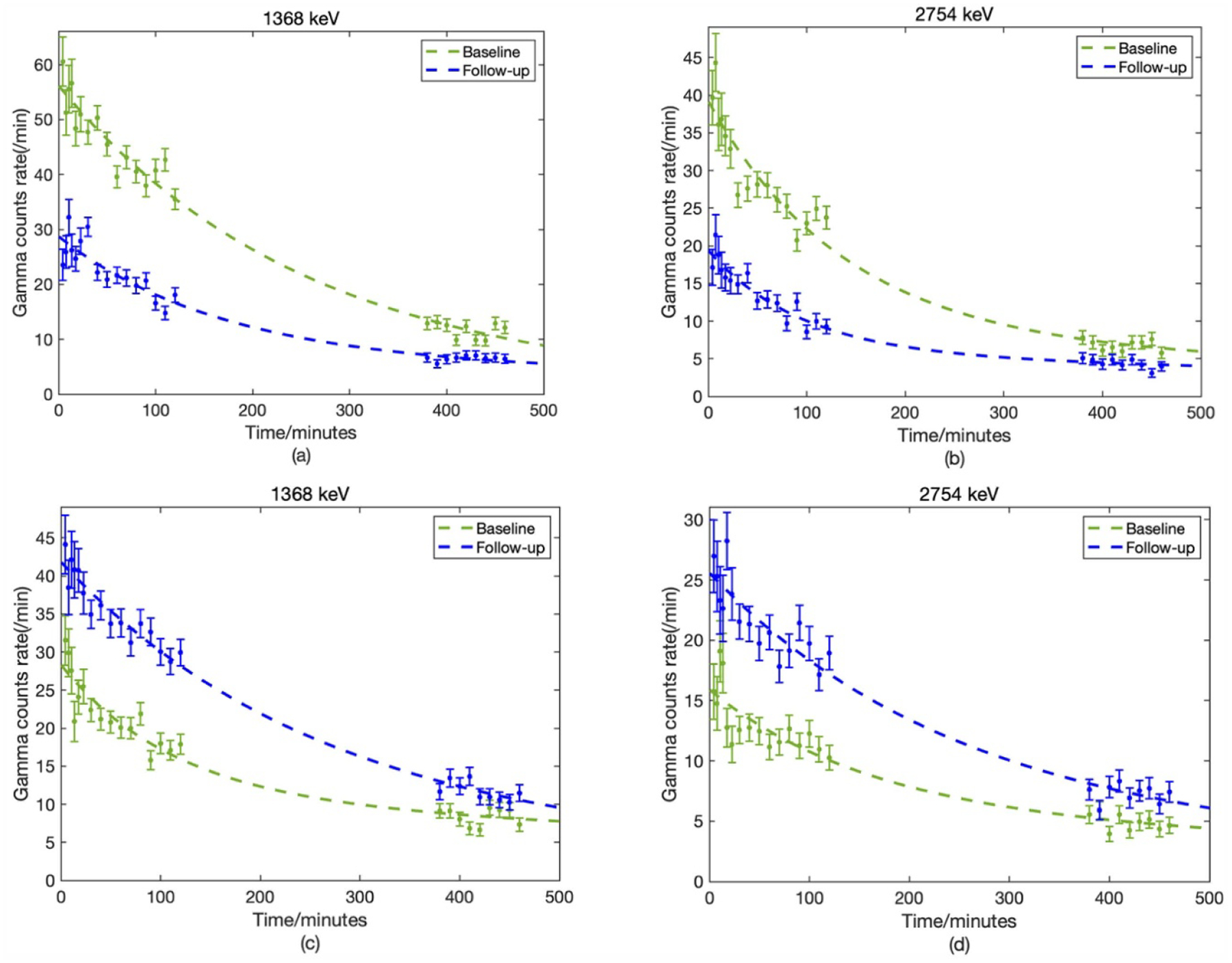
The change of Na signal over time at baseline and after the dietary interventions. (a) and (b) are 1368 and 2754 keV gamma rays biokinetic model fitting for base line and follow-up measurements for one participant after low Na diet. (c) and (d) are another participant’s 1368 and 2754 keV gamma rays biokinetic model fitting for base line and follow-up measurements after high Na diet. How Na changed in different compartments are reflected.

The gradual change of the Na signal over time can be more clearly demonstrated by the total Na signal in the spectrum at different time intervals, fitted to a biokinetic model. The effect of dietary intervention on altering Na content in the two compartments is reflected by the change of both gamma rays (figure 7). At the tail of each measurement, the curve nearly intersects with each other. As discussed above, most counts in the second measurement come from Na in bone. It indicates that the Na in bone will not be greatly affected by the diet intervention. The significant drop at the beginning is caused by fast Na circulation in soft tissue. Nonsignificant change of Na in bone is expected during the early period because there is not sufficient time for Na to be deposited into bone considering it is a rather stable compartment. The results from two gamma rays were combined together to calculate the bone and soft tissue Na concentrations using the inverse-variance weighted method, minimizing the uncertainty. The error bars for concentration was determined based on the counting error for each individual measurement using calibration curves. The error was calculated as two standard deviations. The student *t*-test was used for statistical analysis, and *p* < 0.05 is considered significant difference.

### 3.3. Clinical Na storage findings

**Figure 8.**
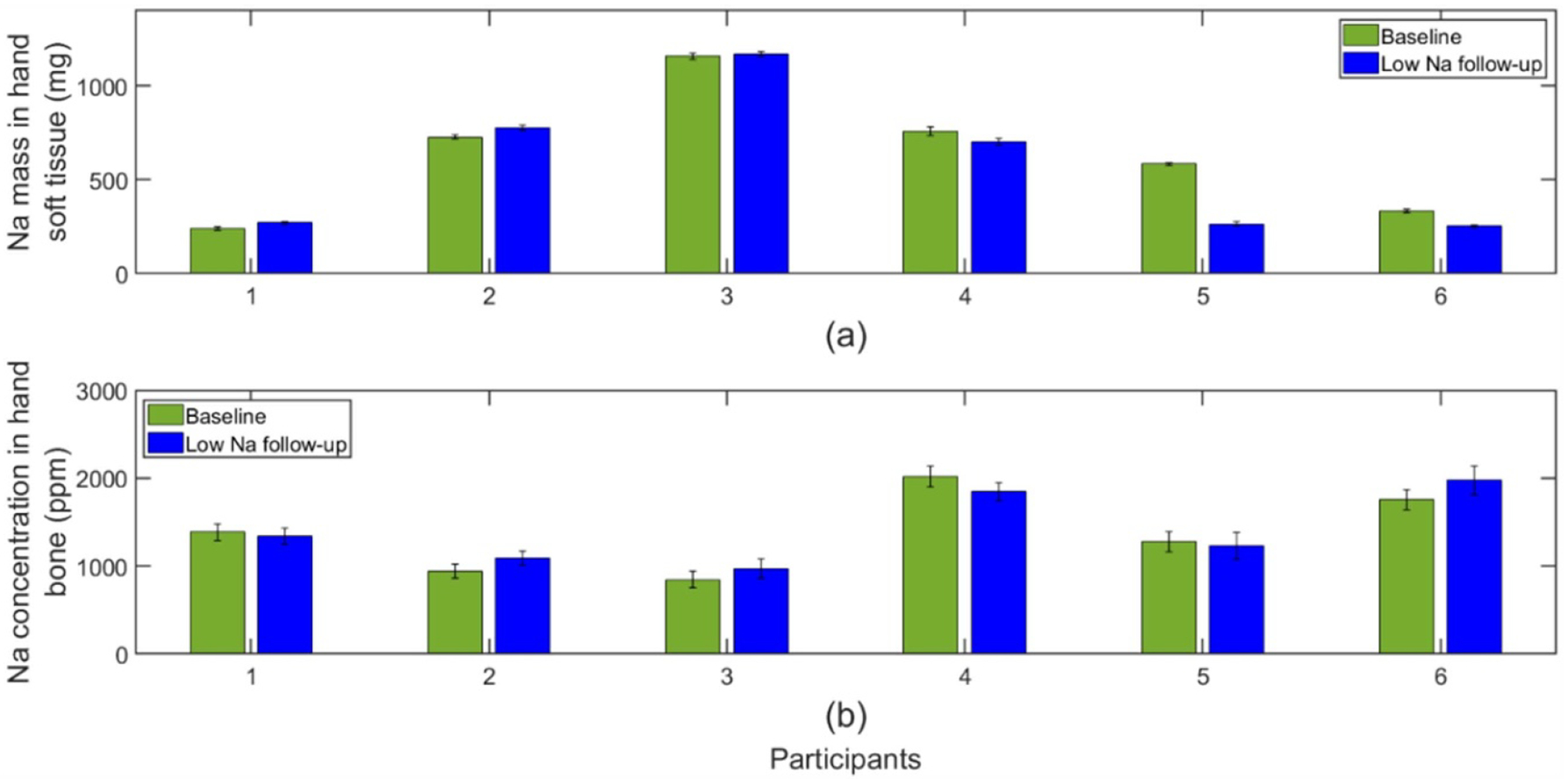
Na level in tissues for participants during the low Na diet intervention for (a) soft tissue (b) bone.

Seven participants completed the low Na dietary intervention and required visits. One participant was excluded due to unexpected much longer feeding caused by delayed scheduling of a clinical visit. The results of remaining participants (3 males and 3 females) with dietary interventions as designed are shown in figure 8. Even though there is no significant difference for Na in soft tissue (*p* = 0.16), some participants presented declined Na in soft tissue after low Na intervention. It can be seen that Na in soft tissue dropped in half of the subjects with low Na diet by amounts ranging from −8% to −55%. However, all bone Na results after the same low Na diet lie within the bounds of uncertainty and did not show changes (*p* = 0.29).

**Figure 9.**
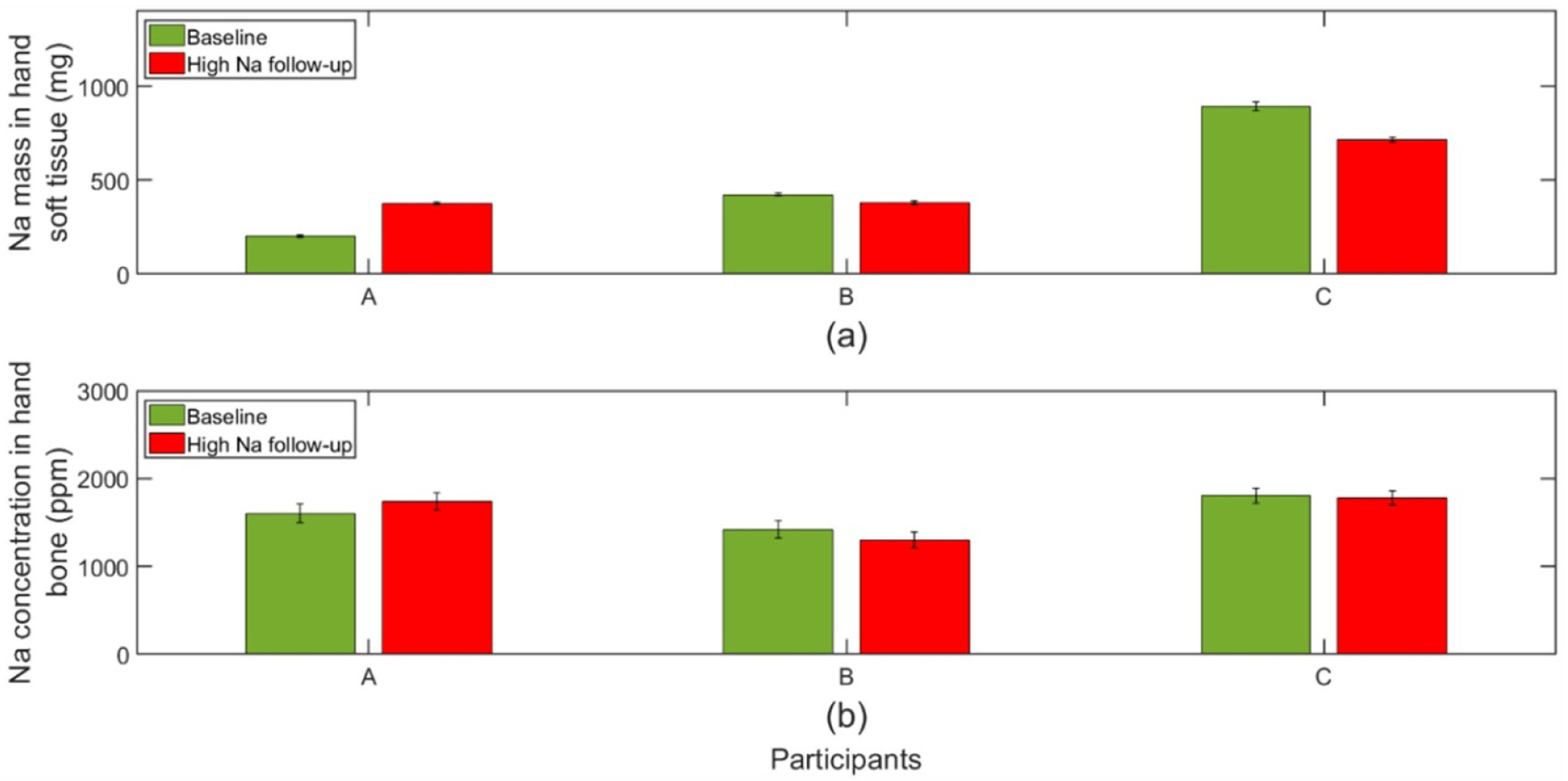
Na level in tissues for participants fed the high Na diet intervention for (a) soft tissue (b) bone.

**Figure 10.**
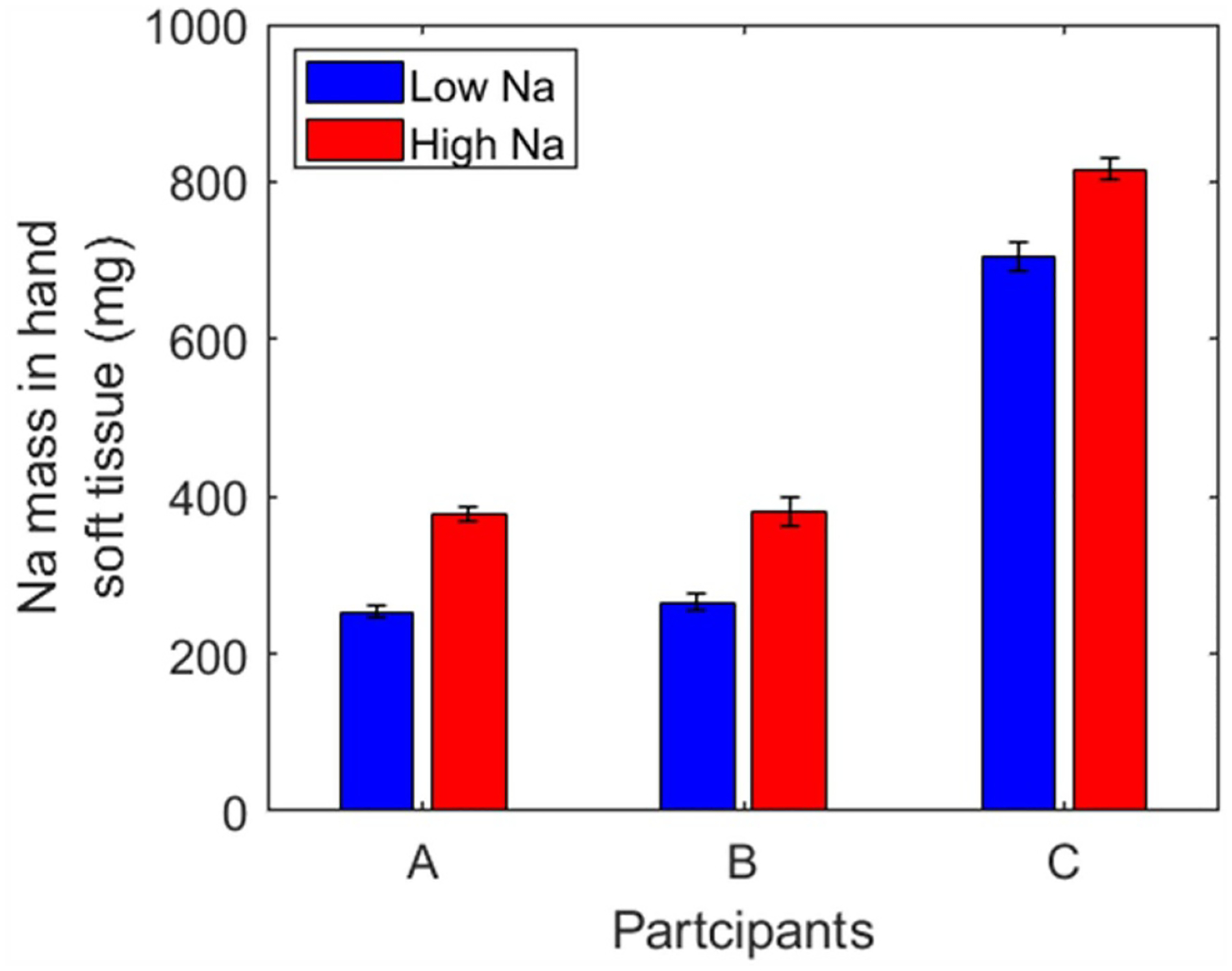
Na level in soft tissue after low and high diet intervention.

Three participants (1 male and 2 females) completed the high Na feeding period and IVNAA measurements. Figure 9 presents Na tissue content before and after being fed a high salt diet. As explained, The Na concentration in bone maintained stable under dietary salt levels (*p* = 0.50). In contrast, there was a response to Na content in soft tissue due to dietary sodium. One participant presented a 88% Na increase in soft tissue, while the other two participants showed a slight drop. It should be noted that there was no diet control during the washout time between the two dietary intervention periods. So, higher daily salt intake could happen during washout period which could lead to larger baseline measurement. This can explain the results of participants B and C. Fortunately, these three participants also completed the low Na diet feeding. By comparing the soft tissue Na after the low-Na and high-Na diets, it is clear that high Na diet increased Na accumulation in soft tissue (figure 10). A significant Na elevation was observed in soft tissue (*p* < 0.01).

## 4. Discussion and future work

The findings from this study underscore the important role that dietary salt plays in influencing soft tissue Na content, which is especially relevant in the context of chronic disease. The body responds quickly to changes in dietary Na intake, with soft tissues responding to dietary changes faster than bone. This is likely because the storage and deposition of minerals into bone are slower processes compared to soft tissue, which has a more immediate response to fluctuations in Na consumption. The study showed that IVNAA can effectively measure Na concentrations in both bone and soft tissue, making it a valuable tool for monitoring how Na intake affects these compartments of the body. Na levels in these tissues can be correlated with blood pressure to open new avenues for investigating the relationship between Na intake and hypertension.

By normalizing the data to ^49^Ca, accuracy of the Na levels in bone was improved. Na concentration in human bones, ranged from 1000 to 2000 ppm in adults. This value offers a reliable benchmark for Na levels in bone, which could be used as a reference for future studies or in clinical settings to evaluate its retention in individuals. However, it is important to note that this concentration may vary with age, bone density, diet, and underlying health conditions.

There are several limitations of this study: (a) the sample size is small. This is the first time bone and soft tissue data have been collected for a nutrition study based on neutron irradiation with dietary interventions. The sample size was lower than planned as the study was interrupted by the COVID pandemic. (b) The feeding period was short. A 2 week intervention might not be efficient to observe significant changes in bone Na levels. Latent variation could be obscured by experimental uncertainties. (c) There are no food restrictions during the washout period. This could impact baseline measurements and potentially interfere with the expected Na alteration patterns from dietary interventions. (d) Phantoms used in this study did not have the same geometry as human irradiated hand. For bone, this inconsistency was cancelled out by Na/Ca normalization. However, Na concentration in soft tissue cannot be calculated accurately because only mass factor was corrected. For example, the patients movements cannot be accounted and will lead to deviations. In future studies, the information for the hand size can be collected for complete correction. (e) The time interval between the two successive gamma ray signal collection was not optimized. A longer time interval would allow for a more accurate estimate, as it would ensure complete removal of Na from the soft tissue by the second measurement.

In the future, we will continue to improve the sensitivity of the system for clinical use. The merit factor of elemental activation over neutron dose can be further increased. In addition, phantoms fabrication and corrections for soft tissue Na estimation can be further refined. These improvements can minimize radiation exposure and enhance measurement accuracy in clinical applications.

## 5. Conclusions

This study measured Na in both soft tissue and bone using IVNAA. Na level changes were reflected in soft tissue as well as in the Na biokinetics curve under two dietary salt interventions. Future work should relate changes in sodium tissue content with disease risk outcomes such as blood pressure to determine their relationship.

## Data Availability

All data that support the findings of this study are included within the article (and any supplementary information files).
